# Self-Assembly Behavior, Aggregation Structure, and the Charge Carrier Transport Properties of S-Heterocyclic Annulated Perylene Diimide Derivatives

**DOI:** 10.3390/molecules29091964

**Published:** 2024-04-25

**Authors:** Haijie Ben, Gaojie Yan, Yulin Wang, Huiming Zeng, Yuechao Wu, Feng Lin, Junhua Zhao, Wanglong Du, Shaojie Zhang, Shijia Zhou, Jingyu Pu, Milan Ye, Haifeng Ji, Liang Lv

**Affiliations:** 1 College of Chemical and Material Engineering, Quzhou University, Quzhou 324000, China; benhaijie@tju.edu.cn (H.B.); csu_lin@163.com (Y.W.); weimingzeng@tom.com (H.Z.); wuyuechao_qzu@163.com (Y.W.); linf2015@126.com (F.L.); qzzjh@qzc.edu.cn (J.Z.); zhangshaojie031026@163.com (S.Z.); yemilan@qzc.edu.cn (M.Y.); 2 Shenzhen Research Institute of Nankai University, Nankai University, Shenzhen 518083, China; 202021501025@stu.hebut.edu.cn; 3 College of Chemical Engineering, Zhejiang University of Technology, Hangzhou 310014, China

**Keywords:** perylene diimide, S-heterocyclic annulated, crystalline structure, charge carrier mobility, structure–function relationship

## Abstract

The construction of high-performance n-type semiconductors is crucial for the advancement of organic electronics. As an attractive n-type semiconductor, molecular systems based on perylene diimide derivatives (PDIs) have been extensively investigated over recent years. Owing to the fascinating aggregated structure and high performance, S-heterocyclic annulated PDIs (SPDIs) are receiving increasing attention. However, the relationship between the structure and the electrical properties of SPDIs has not been deeply revealed, restricting the progress of PDI-based organic electronics. Here, we developed two novel SPDIs with linear and dendronized substituents in the imide position, named linear SPDI and dendronized SPDI, respectively. A series of structural and property characterizations indicated that linear SPDI formed a long-range-ordered crystalline structure based on helical supramolecular columns, while dendronized SPDI, with longer alkyl side chains, formed a 3D-ordered crystalline structure at a low temperature, which transformed into a hexagonal columnar liquid crystal structure at a high temperature. Moreover, no significant charge carrier transport signal was examined for linear SPDI, while dendronized SPDI had a charge carrier mobility of 3.5 × 10^−3^ cm^2^ V^−1^ s^−1^ and 2.1 × 10^−3^ cm^2^ V^−1^ s^−1^ in the crystalline and liquid crystalline state, respectively. These findings highlight the importance of the structure–function relationship in PDIs, and also offer useful roadmaps for the design of high-performance organic electronics for down-to-earth applications.

## 1. Introduction

In a smart society, organic electronic devices represent an exciting field at the intersection of materials, science, and electronics, with potential implications for diverse industries and everyday life [1,2]. For constructing organic electronic devices, p-type and n-type materials are equally important [3]. However, the development of n-type semiconductor materials lags far behind that of p-type semiconductors, greatly limiting the evolution of organic electronic devices [4,5,6]. Facing this dearth, perylene diimide derivatives (PDIs), a kind of promising n-type semiconductors with delectable electron carrier mobilities, have drawn our attention [7,8,9]. Suitably functionalized PDIs exhibit the great advantages of a wide range of UV-visible light absorption, low LUMO energy levels, high molar absorption coefficients, and adjustable energy levels, growing into an important class of molecules for progress in various organic electronics, including organic field-effect transistors, solar cells, and photocatalysts, etc. [10,11,12].

Recently, researchers have gradually realized the potential value of S-heterocyclic annulated PDIs (SPDIs) [13,14]. The additional S⋯O and intermolecular S⋯S interactions contribute to more special-ordered structures of PDI cores, playing a major role in enhancing charge transport [15,16]. Subsequently, SPDIs have always demonstrated significantly higher carrier mobilities compared to the common PDIs or even the O-, N-, and Se-heterocyclic annulated PDIs [17,18]. The above results indicated that SPDIs are a kind of inspired organic electronic devices. It is understood that the carrier mobility is a significant parameter for organic electronic devices [19]. Studying the relationship between the self-assembly behavior and charge carrier transport properties is important to enhancing the properties of SPDIs, but is still lacking.

Usually, the modification of the *N*-positions in PDIs could significantly regulate the self-assembly behavior of PDIs [20,21], as well as the solubility [22] and the film microstructure of PDIs [23]. In order to systematically study the relationship between the self-assembly behavior and the charge carrier transport properties of SPDIs, we designed and synthesized SPDIs with different *N*-substituents—bifurcately linear alkyl chains and dendronized tri-alkoxy phenyl groups, respectively. As the commonly used substituents, the bifurcately linear alkyl chains in the imide position always contributed to better solubility and solution processability than those with the straight ones [24,25], while the tri-alkoxy phenyl groups in the imide position facilitated a better electronic performance [17,26]. To understand the evolutive properties of SPDIs with bifurcately linear alkyl chains and dendronized tri-alkoxy phenyl groups, the self-assembly behavior, aggregated structure, and electrical properties were elaborately investigated and analyzed, and the relationship between the self-assembly behavior and the charge carrier transport properties of the SPDIs was revealed. These results provide a theoretical and experimental foundation for the development and application of this class of materials. 

## 2. Results

### 2.1. The Self-Assembly Behavior of SPDIs in Solution

**Figure 1:**
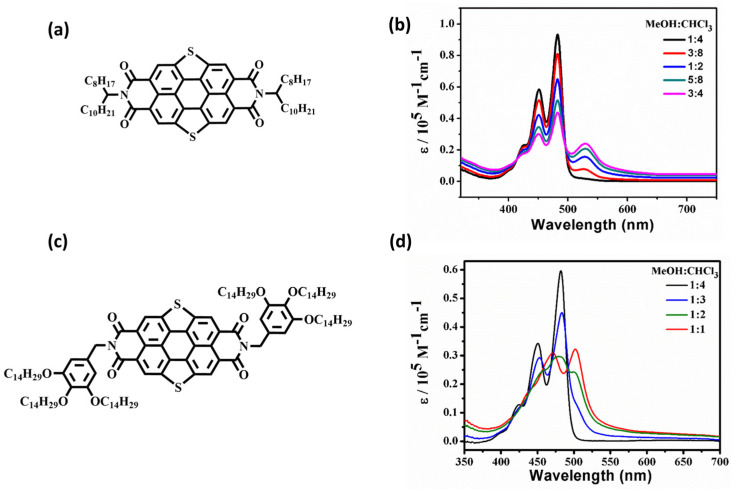
The chemical formulas of (**a**) linear SPDI and (**c**) dendronized SPDI, and the corresponding solvent-dependent UV/Vis spectra of (**b**) linear SPDI in MeOH/CHCl_3_ mixed solvent systems with different ratios from 1/4 to 3/4, (**d**) dendronized SPDI in MeOH/CHCl_3_ mixed solvent systems with different ratios from 1/4 to 1/1 in the concentration of 3 × 10^−5^ mol/L at 25 °C.

Solvation is a significant factor in controlling the self-assembly behavior of aromatic π-π stacking. In order to understand the self-assembly behavior of the as-prepared samples, we performed photophysical studies in a solution, in which CHCl_3_ was a good solvent for linear SPDI and dendronized SPDI, while MeOH was a poor solvent. Figure 1 shows the chemical formulas of the as-prepared samples and the corresponding solvent-dependent UV/Vis absorption spectra in a mixed solvent of CHCl_3_ and MeOH. In a high CHCl_3_ content, well-resolved absorption spectra with typical three vibronic bands could be witnessed for the two PDIs, representing S_0−0_, S_0−1_ and S_0−2_ vibronic transitions, respectively [27]. Additionally, the value of A^0→0^/A^0→1^ at the lowest concentration is greater than 1.6, characteristic of the molecularly dissolved state of typical PDIs [28]. With an increase in the methanol content, the distinct spectral changes could be detected, meaning that the aggregation of both dyes was strongly triggered. In detail, for the linear SPDI (Figure 1b), the intensities of the three absorption peaks in the spectrum were weakened once the MeOH/CHCl_3_ was greater than 1/4, and gradual red shifts could be observed with the increasing titration of MeOH. Moreover, a new peak could be distinguished at a longer wavelength of 537 nm, representing the formation of π–π stacking of parent PDI chromophores, while for dendronized SPDI with the addition of MeOH, more obvious self-assembly behavior could be evidenced (Figure 1d). In addition to the weakened peaks, a new absorption maximum with a substantially smaller *ε* value arises at 502 nm could also be detected. The new absorption maximum was caused by the very strong electronic couplings between PDI skeletons [29], indicating the drastic self-assembly behavior induced by the strong intermolecular π–π interaction force (Figure 1d). This aggregation could also be observed through the tailing phenomenon in the absorption spectra of dendronized SPDI, caused by the light scattering of the self-assembled nanoparticles [17,30]. Correspondingly, the intermolecular distance of dendronized SPDI was closer than that of the linear SPDI, according to the theoretical calculation results (Supplementary Materials: Figure S1), also proving the stronger aggregation in dendronized SPDI.

### 2.2. The Thermal Properties and Self-Assembly Behavior of SPDIs in Solid

**Figure 2:**
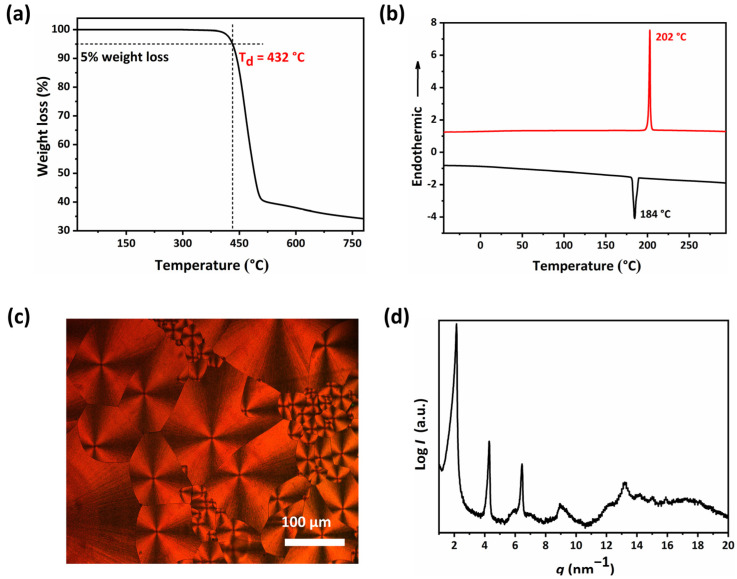
(**a**) The TGA curve and (**b**) DSC cooling and subsequent heating thermal diagrams of linear SPDI with temperature changes at a rate of 10 °C/min, and the arrow in the figure represented the endothermic direction along the y-axis. The typical textures of the linear SPDI film as observed by POM: (**c**) the anisotropic crystals obtained from the slowly decreasing the temperature to 25 °C, and (**d**) 1D SAXD pattern of linear SPDI.

TGA and DSC could be used to investigate the thermal stability and phase behavior of the linear SPDI samples. The decomposition temperature of the linear SPDI reached 432 °C at a rate of 10 °C/min rise, indicating its excellent thermodynamic stability (Figure 2a). The recorded cooling and heating curves revealed the phase transition of linear SPDI, in which the melting and crystallization temperature of the linear SPDI sample was 202 °C and 184 °C, respectively (Figure 2b). The polarized texture of the linear SPDI was recorded using a polarizing optical microscope (POM). By slowly cooling the sample from an isotropic temperature to room temperature, the birefringent textures could be observed (Figure 2c), demonstrating the strong crystallization in the linear SPDI. To gain a deeper understanding of the crystal structure and the self-assembly behavior of the linear SPDI, 1D small-angle X-ray diffraction experiments (SAXD) were conducted. As shown in Figure 2d, distinct diffraction peaks could be observed in both the low-angle and high-angle regions, indicating the formation of a highly ordered crystalline structure. Among them, there are three-order diffraction peaks in the low-angle region of the sample, with a ratio of their q-values as 1:2:3, revealing the formation of a long-range-ordered layered structure in the linear SPDI. 

**Figure 3:**
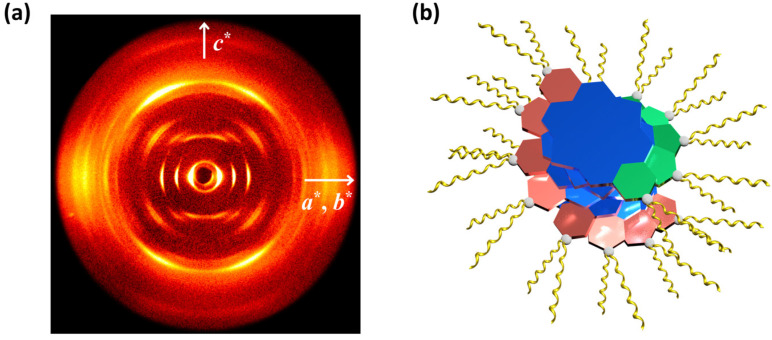
(**a**) The 2D WAXD pattern of the linear SPDI, and the arrow of a*- and b*-axis are assigned to the equator, and the arrow of c*-axis is assigned along the meridian. (**b**) Self-assembly structure model of the linear SPDI and, for clarity, the benzene rings are drawn in different colors.

To further understand the structure and self-assembly behavior of the linear SPDI, 2D wide-angle X-ray diffraction (WAXD) was performed. The linear SPDI sample was cooled from the isotropic melt, then sheared near 200 °C, followed by cooling to room temperature. As shown in Figure 3a, diffraction spots can be observed on the equator, meridian, and interval regions, exhibiting the development of a 3D-ordered crystal structure. The a*- and b*-axis are assigned to the equator, and the c*-axis is assigned along the meridian. Following the procedure for analyzing crystal structures [31,32,33,34], the experimental and calculated unit cell parameters of linear SPDI were obtained as a monoclinic one with dimensions of a = 3.20 nm, b = 2.99 nm, c = 1.11 nm, α = β = 90°, γ = 92.57°. The experimental and calculated diffraction angles and d-spacing values of the crystal lattice were listed in Table S1. Specifically, several diffractions, ranging from 0.37 to 0.48 nm, could be observed in the high-angle region of the meridian (Table S1), suggesting the ordered alignment of the alkyl chains [35,36]. It is worth noting that in the meridian direction, there was a prominent pair of diffractions with a d-spacing of 0.35 nm. Given that this spacing corresponded to the standard π–π stacking distance of the PDI cores, these distinctive arcs revealed the parallel stacking of the PDI cores [37]. Additionally, this spacing was located on the 24th layer of the diffractions, which could be indexed as (033). Such a large c parameter of the linear SPDI indicated that the molecules are rotationally stacked and helically assembled in the supramolecular column, giving a 33-fold helix. During the crystallization process, the free energy should be reduced for the helical packing of both the aromatic cores and alkyl chains. At this stage, a certain degree of rotation of the linear SPDI molecules within the supramolecular columns was induced from the mismatch in crystallization thickness between the aromatic cores and alkyl chains, ultimately leading to the formation of complex supramolecular structures. According to the above analysis, the self-assembly structure model of the linear SPDI was designed (Figure 3b). In the figure, the chromophores of the linear SPDIs stacked one above the other with certain angle rotation, and finally assembled into the helical column. In order to further prove the results, we performed the high-resolution scanning tunneling microscopy (STM) to directly observe the self-assembly of the linear SPDI on the surface. As shown in Figure S2, the unit cell parameters were measured to be a = 3.20 ± 0.1 nm, b = 2.83 ± 0.1 nm, γ = 93.63 ± 1.0°, presenting the monoclinic lattice, which corresponded to the results obtained in the XRD patterns. 

**Figure 4:**
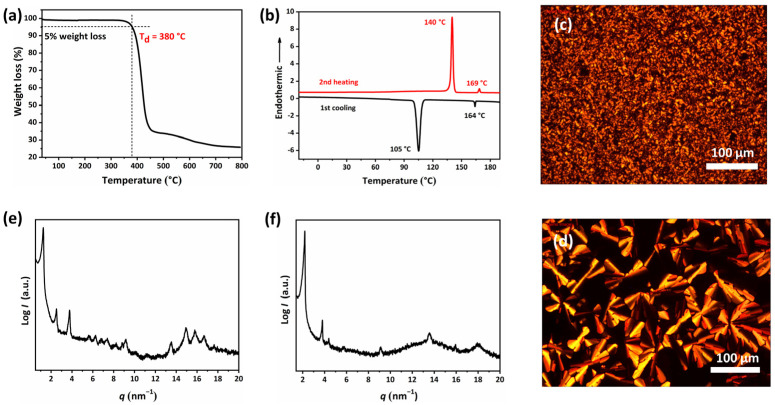
(**a**) The TGA curve and (**b**) DSC cooling and subsequent heating thermal diagrams of the dendronized SPDI with temperature changes at a rate of 10 °C/min, and the arrow in the figure represented the endothermic direction along the y-axis. The typical textures of the linear SPDI film, as observed by POM: (**c**) the anisotropic crystalline phase obtained from the slowly decreasing the temperature to 25 °C, and (**d**) the liquid crystalline phase obtained at 150 °C. The 1D SAXD patterns of (**e**) the crystalline state at room temperature and (**f**) the liquid crystalline state for the dendronized SPDI at 150 °C.

Similarly, we investigated the thermal behavior of the dendronized SPDI sample by TGA and DSC experiments. In the TGA experiment (Figure 4a), the decomposition temperature of the dendronized SPDI could be up to 380 °C at the rate of 10 °C/min rise, exhibiting excellent thermal stability. And, in the DSC experiment (Figure 4b), with a heating and cooling rate of 10 °C/min, two endothermic peaks were located at 140 °C and 169 °C, while two exothermic peaks were located at 105 °C and 164 °C, demonstrating that the dendronized SPDI formed a crystalline structure at a low temperature and a liquid crystalline structure at a relatively high temperature. Interestingly, the self-assembly changes of the dendronized SPDI during the heating and cooling process could be observed in POM. Figure 4c represented the obvious anisotropy of the dendronized SPDI at room temperature in a polarized texture. Once the temperature was elevated to 150 °C, a typical dendritic texture could be detected (Figure 4d), verifying the formation of a columnar liquid crystal structure. To better understand the molecular arrangement of the dendronized SPDI, we conducted further experiments using 1D SAXD and 2D WAXD. Figure 4e,f shows the 1D SAXD curves of the dendronized SPDI at 25 °C and 150 °C, respectively. It could be observed that at 25 °C, the dendronized SPDI exhibited distinct diffraction peaks in both the low-angle and high-angle regions, indicating a high degree of crystalline order. In the low-angle region, there were three diffraction peaks with a ratio of q values of 1:2:3, displaying the presence of a long-range-ordered layered structure. At 150 °C, significant changes occurred in the 1D SAXD pattern, where the sharp diffraction peaks, corresponding to the crystallization of alkyl chains, transformed into diffused scattering patterns in the high-angle region. While in the low-angle region, the ratio of q values became 1:$\sqrt{3}$:2, presenting that the dendronized alkyl side chains could gradually melt at a high temperature, promoting the columnar arrangement of SPDI aromatic cores and further assembling into a hexagonal columnar liquid crystal structure. 

**Figure 5:**
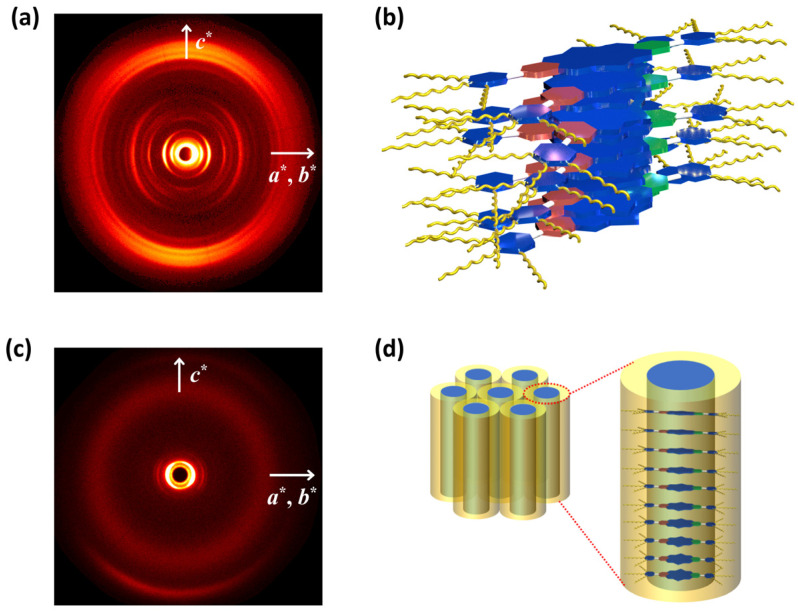
(**a**) 2D WAXD patterns in the crystalline state of the dendronized SPDI at room temperature, and the arrow of a*- and b*-axis are assigned to the equator, and the arrow of c*-axis is assigned along the meridian, and (**b**) the corresponding model of the 3D supramolecular structure with benzene rings are drawn in different colors for clarity. (**c**) 2D WAXD patterns in the liquid crystalline state of the dendronized SPDI at 150 °C, and the arrow of a*- and b*-axis are assigned to the equator, and the arrow of c*-axis is assigned along the meridian, and (**d**) schematic illustration of the supramolecular structure of the hexagonal column structure of the dendronized SPDI.

To specifically analyze the crystal structure of the dendronized SPDI, we used mechanical external force to orient the sample and obtained 2D WAXD spectra in both the crystalline and liquid crystal states, as shown in Figure 5. At 25 °C, the direction of the external shear force was parallel to the meridian direction of the diffraction pattern, while the X-ray incident direction was perpendicular to the shear direction of the sample (Figure 5a). The crystal plane spacing, corresponding to the diffraction arcs in the 2D WAXD, matched well with the 1D SAXD diffraction curves. Note that the 2D WAXD patterns could provide more detailed diffraction curves and crystalline information than the 1D SAXD patterns. In detail, the distinct diffraction arcs in both the low-angle and wide-angle regions could be observed, proving the development of a 3D-ordered crystal structure of dendronized SPDI at room temperature. It should be stated that the diffraction arcs in the high-angle region corresponded to the crystallization peak of the alkyl side chains, proving the crystalline state of the alkyl chains at a low temperature [31,32]. Additionally, the observed diffractions, not only on the equator and meridian, but also in the quadrants, revealed the ordered crystalline phase of perylene. On the meridian, there was a strong diffraction arc with d-spacing of 0.37 nm in the high-angle region, corresponding to the π-π interaction of the SPDI aromatic cores, proving the cofacial stacking of the perylene skeletons [33]. The corresponding model of the three-dimensional supramolecular structure of the dendronized SPDI at 25 °C is exhibited in Figure 5b. In the figure, the chromophores of the dendronized SPDIs are crosswise stacked one above the other to refrain from the steric effect of dendronized alkyl chains, and finally assembled into the ordered column. Comparatively, upon heating up to 150 °C (Figure 5c), the 2D XRD pattern was significantly changed. Compared to Figure 5a, the diffraction arcs of the alkyl chains were fewer and evolved into diffusion arcs, presenting the gradual disruption of the long-range-ordered crystal structure and the increased mobility of the alkyl side chains. Moreover, the diffraction arc with d-spacing of 0.37 nm in the high-angle region still existed, while becoming weaker. This phenomenon represented that the continuous stacking of the SPDI aromatic cores had not been destroyed, but that the intermolecular distance was elongated. The above results indicated that the carrier transport properties in the liquid crystalline state might be lower than that in the crystalline state of the dendronized SPDI. In the equatorial direction, the ratio of the lattice spacings corresponding to the three diffraction arcs in the small-angle region was 1:$\sqrt{3}$:2, which was consistent with the results of the 1D SAXD diffraction patterns, indicative of the further arrangement of the supramolecular columns formed by the continuous stacking of the SPDI aromatic cores into a hexagonal structure. The corresponding model of the 3D supramolecular structure of the dendronized SPDI at 150 °C is exhibited in Figure 5d. Because of the melt of the alkyl chains, the chromophores of the dendronized SPDIs were orderly stacked one above the other and were successively assembled into a hexagonal column structure (Figure 5d).

### 2.3. The Charge Carrier Transport Properties of SPDIs

**Figure 6:**
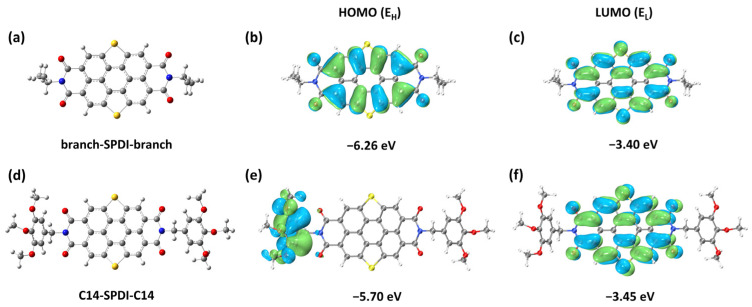
(**a**,**d**) Optimized geometry, (**b**,**e**) HOMO, and (**c**,**f**) LUMO energy levels of the corresponding SPDIs, respectively (for the purpose of the calculation, the chain length was taken as methyl).

DFT quantum mechanical calculations were performed to demonstrate the molecular conformation and front molecular orbitals (HOMO and LUMO) of the SPDIs and further, to evaluate the charge carrier transport capabilities of the SPDIs. The energy-minimized geometries for the SPDIs were obtained by DFT (Figure 6a,d). As observed, the perylene skeletons kept planar even after bay annulation. Then, the HOMO-LUMO energy levels were also calculated to visualize the distribution of the electron clouds and the degree of delocalization in the molecular orbitals. As shown in Figure 6b,c for the linear SPDI, the HOMO-LUMO energy levels were predominantly spread on the central ring, but not on the alkyl chains. However, the HOMO levels of the dendronized SPDI were mainly located on the peripheral benzene moieties, while its LUMO frontier molecular orbitals were mainly distributed on the perylene skeletons (Figure 6e,f). Comparatively, the HOMO-LUMO energy levels of the dendronized SPDI displayed stronger delocalization than the linear SPDI, indicating the better charge carrier transport capabilities of the dendronized SPDI [38].

In order to experimentally evaluate the charge carrier transport properties of SPDIs, we performed the time-of-flight method (TOF), which placed the sample between two electrodes. One of the electrodes was irradiated by transient light to generate charge carriers, migrating to the other electrode to obtain a transient photocurrent curve under the drive of the external electric field. The charge carrier mobilities could be mathematically calculated as the following function:

*μ* = *l*·*t_r_*^−1^·*E*^−1^ (1)

where *μ* represented the charge carrier mobility, *l* was the thickness of the photoconductive film, *t_r_* was the transit time, and *E* was the electric field strength.

**Figure 7:**
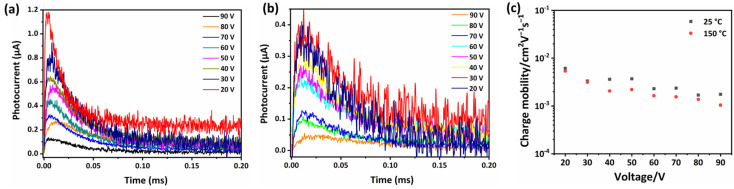
(**a**) Typical TOF transits for the crystalline state of the dendronized SPDI at 25 °C; (**b**) Typical TOF transits for the liquid crystalline state of the dendronized SPDI at 150 °C; (**c**) the voltage dependence of hole mobilities of the dendronized SPDI at room temperature and at 150 °C.

Significant charge carrier transport signals were observed for the dendronized SPDI sample, while no obvious charge carrier transport signal was detected for the linear SPDI (Figure 7). These results indicated the better charge carrier transport capabilities of the dendronized SPDI, in agreement with the DFT calculations. In detail, for the dendronized SPDI, the charge carrier mobilities were detected to be approximately 3.5 × 10^−3^ cm^2^ V^−1^ s^−1^ in the crystalline state at 25 °C, while it was 2.1 × 10^−3^ cm^2^ V^−1^ s^−1^ in the liquid crystalline state at 150 °C. The values of charge carrier mobilities of the dendronized SPDI were relatively high, compared to that of some other PDIs detected using the TOF technique [39,40,41]. The lowered charge carrier mobilities in the liquid crystalline state of the dendronized SPDI could be attributed to the relatively larger intermolecular distance and less ordered 3D assembly structure than that in the crystalline state, which lowered the π–π interaction of the SPDI aromatic cores [42,43].

## 3. Materials and Methods

### 3.1. Materials

1,6,7,12-tetrachloroperylene tetracarboxylic acid dianhydride (Beijing HWRK company, Beijing, China, 98%), nonadecan-9-amine (Beijing HWRK company, 95%), tetrakis(triphenylphosphine)palladium(0) (J&K Scientific Company, Beijing, China, 99.8%), and bis(tri-n-butyltin)sulfide (Gelest, Morrisville, PA, USA, 97%) were used as received. Toluene (AR), trichloromethane (AR), dichloromethane (AR) and methyl alcohol (AR) were purchased from the Yuanli company (Tianjin, China), and were dried through molecular sieves and activated alumina (Heowns, Tianjin, China), and then were distilled by the standard procedures.

### 3.2. Equipment and Characterization

The ^1^H NMR spectra measurements were conducted at ambient conditions, employing the Bruker Avance spectrophotometer with an operating frequency of 400 MHz at room temperature. Chemical shifts (δ) were reported at a standardized temperature of 298 K, utilizing a minute aliquot of tetramethylsilane (TMS) as the internal standard to ensure precision. Thermogravimetric analysis (TGA) was executed, utilizing a TA SDTQ-600 apparatus under a nitrogenous shield, spanning an extensive temperature range from ambient up to 800 °C. DSC metrics were obtained via a TA Q100 apparatus, integrated with a mechanical refrigeration unit. The temperature and heat flow were calibrated using standard materials such as ben-zoic acid and indium. The structural examination via 1D XRD of the air-dried specimen was performed on a SAXSess setup (Anton Paar), incorporating a Kratky block-collimation system which augments high-flux small-angle X-ray scattering capabilities. An imaging plate detector was adopted, facilitating the concurrent assessment of small-angle and wide-angle X-ray scattering phenomena across the specimen, covering a q-range cardinality from 0.06 to 29 nm^−1^ (q = 4πsinθ/λ, where λ represents the wavelength of 0.1542 nm and 2θ is the scattering angle). The acquisition of 2D wide-angle X-ray diffraction (WAXD) patterns was accomplished in a transmission stance using a Bruker D8 Discover diffractometer outfitted with a Vantec500 as the 2D detector system. The oriented sample, prepared by mechanical shearing, was mounted on the sample stage, which incorporated a temperature control unit. The 2D XRD patterns were obtained through a transmission mode, with the X-ray incident beam aligned perpendicular to the shearing direction. The UV/Vis absorption spectra were meticulously captured employing an Agilent Technologies Cary 300 spectrophotometer to ascertain optical properties. The crystal morphologies were observed using an Olympus (HB-2) polarized optical microscopy (POM). Elemental analysis (C, H, N) was performed on an Elementar Vario EL CUBE elements analyzer. For the scanning tunneling microscopy (STM) observations, a droplet of solution, containing one of the three mentioned molecules, was initially placed on the surface of freshly prepared highly oriented pyrolytic graphite (HOPG, grade ZYB, Vecco, Plainview, NY, USA). Subsequently, the tip was immersed in the liquid film at the solid–liquid interface to capture images of the formed self-assembled monolayers (SAMs) with atomic resolution. The experiments were conducted using a Pico-SPM (Molecular Imaging/Agilent Technology, Santa Clara, CA, USA) in constant-current mode under atmospheric conditions at room temperature. Microscopic imaging was repeated multiple times for each sample across different surface areas to mitigate any potential artificial errors.

### 3.3. Theoretical Calculation Methods

The configurations of the linear and dendronized SPDIs and their dimer structures were all optimized under the framework of density of functional theory (DFT) with B3LYP functional [44,45,46], DFT-D3 dispersion correction method [47,48] and 6–31G(d) basis set [49]. The geometries of these molecules in their anion and cation states were also optimized using the same method. In order to obtain the electron energy with higher accuracy, single point calculations for these optimized structures with M062x functional [50] and def2-TZVP basis set were performed. The reorganization energy was calculated using Nelsen’s four-point method, according to Marcus theory. All these DFT calculations were performed using the Gaussian 16 program suite. The visualization of the frontier molecular orbitals was rendered using the Visual Molecular Dynamic program (VMD) [51].

### 3.4. The Synthesis of Linear SPDI

**Scheme 1:**
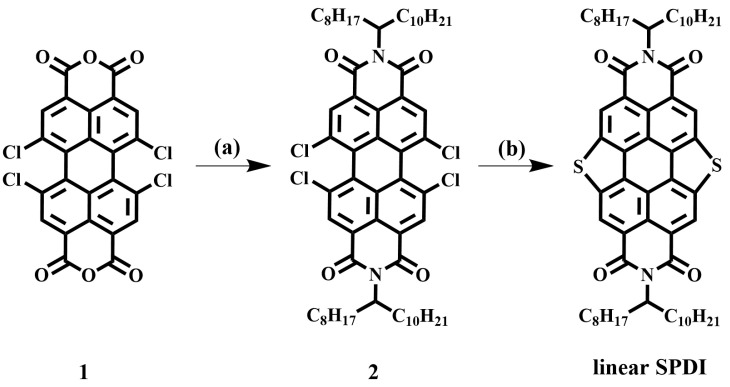
The synthesis process of the linear SPDI: (**a**) nonadecan-9-amine, toluene, 100 °C; (**b**) Pd(PPh_3_)_4_, Bu_3_SnSSnBu_3_, toluene, 100 °C.

Synthesis of compound **2** in Scheme 1: Prepare a 250 mL round-bottom flask equipped with a reflux condenser. Add 1,6,7,12-tetrachloro-3,4,9,10-tetraoxa-perylene-2,11-dione (2.00 g, 3.77 mmol), nonadecan-9-amine (2.45 g, 8.65 mmol), and 50 mL of toluene into the round-bottom flask. Stir vigorously to ensure a homogeneous mixture. Under a nitrogen atmosphere, heat the reaction mixture to 100 °C and reflux for 6 h. After a complete reaction, cool to room temperature and evaporate the solvent. Purify the crude product by column chromatography (dichloromethane:petroleum ether, 1:9) to obtain a red solid (yield: 98%). ^1^H NMR (400 MHz, CDCl_3_) δ (TMS, ppm): 8.70 (s, 4H, perylene ring), 4.15 (d, 4H), 2.01 (m, 2H), 1.27 (m, 64H), 0.88 (m, 12H). Anal. calcd for C_64_H_86_Cl_4_N_2_O_4_ (%): C 70.57, H 7.96, N 2.57; found: C 70.61, H 7.94, N 2.56.

Synthesis of linear SPDI in Scheme 1: Prepare a Schlenk flask equipped with a reflux condenser. Add compound **2** (2.00 g, 1.83 mmol) and tetrakis(triphenylphosphine)palladium (0.57 g, 0.49 mmol) into the Schlenk flask. Connect a double manifold and evacuate and fill with nitrogen gas three times to create a nitrogen atmosphere in the system. Inject bis(tributyltin) sulfide (2.46 g, 4.00 mmol) and 25 mL of toluene using a dry syringe in the system. Heat the reaction mixture to 100 °C and reflux for 10 h. After a complete reaction, cool and remove the solvent under reduced pressure to obtain the crude product. Purify the product by column chromatography (dichloromethane:petroleum ether, 1:2) to yield a yellow solid (yield: 72%). ^1^H NMR (400 MHz, CDCl_3_) δ (TMS, ppm): 9.34 (s, 4H), 4.30 (d, 4H), 2,10 (m, 2H), 1.39 (m, 64H), 0.82 (m, 12H). Anal. calcd for C_64_H_86_N_2_O_4_S_2_ (%): C 75.99, H 8.57, N 2.77; found: C 76.11, H 8.54, N 2.76.

### 3.5. The Synthesis of Dendronized SPDI

**Scheme 2:**
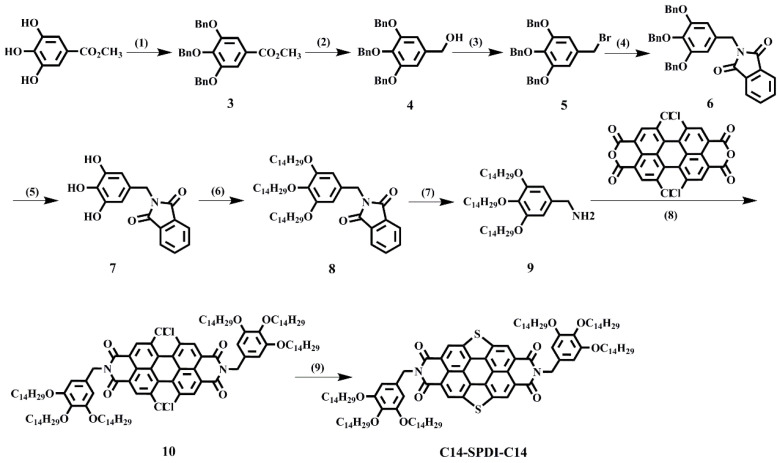
The synthesis process of dendronized SPDI: (1) K_2_CO_3_, Benzyl chloride, DMF, 75 °C; (2) LiAlH_4_, anhydrous THF; (3) anhydrous THF, PPh_3_, NBS, 25 °C; (4) potassium phthalimide, anhydrous DMF, 80 °C; (5) MeOH:CH_2_Cl_2_ = 1:2, Pd/C, hydrogen atmosphere, 80 °C; (6) K_2_CO_3_, degassed DMF, n-tetradecyl bromide, 70 °C; (7) EtOH, hydrazine hydrate, refluxed; (8) 1,6,7,12-tetrachloro-3,4,9,10-tetraacetyldodecane, toluene, refluxed; (9) bis(tributyltin) sulfide, tetrakis(triphenylphosphine)palladium, 100 °C.

Synthesis of methyl 3,4,5-tris(benzyloxy)benzoate (compound **3**) in Scheme 2 [52]: To a suspension of K_2_CO_3_ (31.10 g, 225.0 mol) in degassed DMF (70 mL), methyl 3,4,5-trihydroxybenzoate (9.71 g, 50.0 mmol) was added at room temperature. Benzyl chloride in degassed DMF (30 mL) was slowly introduced, and the reaction mixture was stirred at 75 °C for 12 h. The resulting mixture was poured into cold water, and the solid precipitate was collected via vacuum filtration. The crude solid was dissolved in CH_2_Cl_2_ and was passed through a basic Al_2_O_3_ column, eluted using CH_2_Cl_2_. The solvent was then evaporated under a reduced pressure. Precipitation from CH_2_Cl_2_ in MeOH provided the pure product as a white solid (20.93 g, 92%). ^1^H NMR (400 MHz, CDCl_3_) δ (TMS, ppm): 7.47–7.41 (m, 4H), 7.41–7.29 (m, 10H), 7.28–7.21 (m, 3H), 5.13 (s, 4H), 5.11 (s, 2H), 3.88 (s, 3H).

Synthesis of (3,4,5-tris(benzyloxy)phenyl)methanol (compound **4**) in Scheme 2 [52]: To a suspension of LiAlH_4_ (2.00 g, 52.8 mmol) in anhydrous THF (100 mL) at 0 °C under an argon atmosphere, a solution of methyl 3,4,5-tris(benzyloxy)benzoate (16.00 g, 35.2 mmol) in 40 mL of THF was added dropwise. Once the addition was complete, the reaction mixture was stirred at 25 °C for 2 h. The reaction mixture was then quenched by the sequential addition of 2 mL of H_2_O, 2 mL of 15% NaOH, and 6 mL of H_2_O. The granular salts were filtered and washed with THF. The solvent was evaporated completely, and the remaining solid was purified by passing through a column of basic Al_2_O_3_ using CH_2_Cl_2_ as the eluent. This process provided the product as a white solid (14.81 g, 99%). ^1^H NMR (400 MHz, CDCl_3_) δ (TMS, ppm): 7.46–7.39 (m, 6H), 7.36 (t, *J* = 7.4 Hz, 4H), 7.33–7.29 (m, 2H), 7.28–7.24 (m, 3H), 6.66 (s, 2H), 5.10 (s, 4H), 5.04 (s, 2H), 4.57 (d, *J* = 5.9, 2H), 1.66 (t, *J* = 6.0, 1H).

Synthesis of (((5-(Bromomethyl)benzene-1,2,3-triyl)tris(oxy)) tris (methylene))tribenzene (compound **5**) in Scheme 2 [53]: A solution of (3,4,5-tris(benzyloxy)phenyl)methanol (7.00 g, 16.41 mmol) in anhydrous THF (35 mL) was prepared. To this solution, freshly recrystallized PPh_3_ (5.17 g, 19.70 mmol) was added. The reaction mixture was stirred for 5 min. NBS (3.51 g, 19.70 mmol) was added in a single portion, and the reaction was stirred at room temperature under nitrogen for 2 h. The precipitation of the reaction mixture in MeOH resulted in the isolation of the pure product as a white solid (7.10 g, 88%). ^1^H NMR (400 MHz, CDCl_3_) δ (TMS, ppm): 7.45–7.30 (m, 12H), 7.29–7.23 (m, 3H), 6.69 (s, 2H), 5.10 (s, 4H), 5.04 (s, 2H), 4.40 (s, 2H).

Synthesis of 2-(3,4,5-Tris(benzyloxy)benzyl)isoindoline-1,3-dione (compound **6**) in Scheme 2 [54]: A suspension of potassium phthalimide (5.10 g, 27.5 mmol) in anhydrous DMF (60 mL) was prepared, and (((5-(bromomethyl)benzene-1,2,3-triyl)tris(oxy))tris(methylene)) tribenzene (14.80 g, 30.24 mmol) was added at room temperature. The reaction mixture was stirred at 80 °C for 12 h. After cooling to room temperature, the reaction mixture was poured into a solution of NaOH. The resulting mixture was extracted with ethyl acetate (3 times), and the organic layer was subsequently washed with water, brine, dried over MgSO_4_, and concentrated. The precipitation from CH_2_Cl_2_ in MeOH afforded the pure product as a white solid (15.30 g, 95%). ^1^H NMR (400 MHz, CDCl_3_) δ (TMS, ppm): 7.85 (dd, 2H), 7.72 (dd, 2H), 7.40 (dd, 6H), 7.34–7.29 (m, 4H), 7.29–7.22 (m, 5H), 6.76 (s, 2H), 5.09 (s, 4H), 4.99 (s, 2H), 4.72 (s, 2H).

Synthesis of 2-(3,4,5-Trihydroxybenzyl)isoindoline-1,3-dione (compound **7**) in Scheme 2 [54]: Into a solution of 2-(3,4,5-tris(benzyloxy)benzyl)isoindoline-1,3-dione (6.00 g, 10.80 mmol) in a mixture of MeOH:CH_2_Cl_2_ = 1:2 (90 mL), Pd/C (0.20 g) was added. The reaction flask was subjected to a vacuum and filled with hydrogen three times. The reaction mixture was stirred at room temperature under a hydrogen atmosphere for 12 h. The catalyst was removed by filtration through Celite^®^, and the solvent was evaporated, resulting in the formation of a pale green solid product (3.08 g, 100%), which was deemed pure enough and used without further purification. ^1^H NMR (400 MHz, DMSO-d6) δ (TMS, ppm): 8.82 (s, 2H), 8.01 (s, 1H), 7.95–7.80 (m, 4H), 6.23 (s, 2H), 4.51 (s, 2H).

Synthesis of 2-(3,4,5-Tris(tetradecyl)benzyl)isoindoline-1,3-dione (compound **8**) in Scheme 2 [55]: A suspension of K_2_CO_3_ (2.18 g, 15.78 mmol) in degassed DMF (15 mL) was prepared. To this suspension, 2-(3,4,5-trihydroxybenzyl)isoindoline-1,3-dione (1.00 g, 3.51 mmol) and n-tetradecyl bromide (2.92 g, 10.52 mmol) were added. The reaction mixture was stirred at 70 °C under a nitrogen atmosphere for 12 h. Afterwards, the reaction was cooled to room temperature and was precipitated in water. The resulting solid was collected by filtration and air-dried. The crude product was then passed through a column of basic Al_2_O_3_ using CH_2_Cl_2_ as the eluent. The solvent was evaporated to a minimal amount, and the product was precipitated in MeOH, resulting in the formation of a white solid. Yield: 2.72 g (91%). ^1^H NMR (400 MHz, CDCl_3_) δ (TMS, ppm): 7.84 (dd, 2H), 7.70 (dd, 2H), 6.65 (s, 2H), 4.72 (s, 2H), 3.95 (t, 4H), 3.89 (t, 2H), 1.79–1.73 (m, 4H), 1.73–1.67 (m, 2H), 1.49–1.40 (m, 6H), 1.37–1.20 (m, 48H), 0.90–0.86 (m, 9H).

Synthesis of (3,4,5-tris(tetradecyl)phenyl)methanamine (compound **9**) in Scheme 2 [55]: Into a solution of 2-(3,4,5-Tris(tetradecyl)benzyl)isoindoline-1,3-dione (2.00 g, 2.53 mmol) in absolute EtOH (35 mL), 64% hydrazine hydrate (1.25 mL, 25.3 mmol) was added in a nitrogen atmosphere and refluxed for 12 h. The reaction mixture was allowed to cool to room temperature. The white precipitate was removed by filtration and washed with Et_2_O. The solvent was evaporated to give the product, which was used for the next step without purification. ^1^H NMR (400 MHz, CDCl_3_) δ (TMS, ppm): 6.51 (s, 2H), 3.97 (t, 4H), 3.92 (t, 2H), 3.78 (s, 2H), 1.82–1.76 (m, 4H), 1.76–1.70 (m, 2H), 1.50–1.42 (m, 6H), 1.38–1.23 (m, 48H), 0.88 (t, 9H).

Synthesis of compound **10** in Scheme 2: Using 1,6,7,12-tetrachloro-3,4,9,10-tetraacetyldodecane (0.27 g, 0.51 mmol), (3,4,5-tris(tetradecyl)phenyl) methanamine (0.78 g, 1.04 mmol) in refluxing toluene (30 mL) for 6h, followed by column separation on silica gel (dichloromethane:petroleum ether, 1:9) to obtain compound **10**. ^1^H NMR (400 MHz, CDCl_3_) δ (TMS, ppm): 8.43 (d, 4H), 8.20 (d, 4H), 6.87 (s, 4H), 5.27 (s, 4H), 4.03 (t, 8H), 3.91 (t, 4H), 1.85–1.76 (m, 8H), 1.75–1.67 (m, 4H), 1.53–1.40 (m, 12H), 1.39–1.17 (m, 112H), 0.94–0.79 (m, 18H).

Synthesis of dendronized SPDI in Scheme 2: Prepare a Schlenk flask equipped with a reflux condenser. Add compound **10** (3.62 g, 1.83 mmol) and tetrakis(triphenylphosphine)palladium (0.57 g, 0.49 mmol) into the Schlenk flask. Connect a double manifold and evacuate and fill with nitrogen gas three times to create a nitrogen atmosphere in the system. Inject bis(tributyltin) sulfide (2.46 g, 4.00 mmol) and 25 mL of toluene using a dry syringe into the system. Heat the reaction mixture to 100 °C and reflux for 10 h. After a complete reaction, cool and remove the solvent under reduced pressure to obtain the crude product. Purify the product by column chromatography (dichloromethane:petroleum ether, 1:2) to yield a yellow solid (yield: 75%). ^1^H NMR (400 MHz, CDCl_3_) δ (TMS, ppm): 9.31 (s, 4H), 6.95 (s, 4H), 5.47 (s, 4H), 4.04 (t, 8H), 3.89 (t, 4H), 1.77 (m, 8H), 1.69 (m, 4H), 1.55 (m, 8H), 1.45 (m, 12H), 1.22 (m, 112H), 0.86 (m, 18H). Anal. calcd for C_64_H_86_N_2_O_4_S_2_ (%): C 76.92, H 9.84, N 1.47; found: C 76.95, H 9.81, N 1.45.

## 4. Conclusions

In this paper, we designed and synthesized two novel PDI derivatives: linear SPDI and dendronized SPDI with bifurcately linear and the dendronized SPDI chains in the imide positions of SPDI. The thermal stability and phase transition and the self-assembly behavior were systematically investigated. Furthermore, the charge carrier transport performance of the samples was detected using TOF methods. Experimental results demonstrated that the linear SPDI formed a long-range-ordered crystalline structure based on helical supramolecular columns, while the dendronized SPDI with longer alkyl side chains formed a 3D-ordered crystalline structure at low temperatures, which was transformed into a hexagonal columnar liquid crystal structure as the temperature increased. It was found that no significant charge carrier transport signal was examined for thed linear SPDI, while the dendronized SPDI had a charge carrier mobility of 3.5 × 10^−^^3^ cm^2^ V^−^^1^ s^−^^1^ and 2.1 × 10^−^^3^ cm^2^ V^−^^1^ s^−^^1^ in the crystalline and liquid crystalline state, respectively, indicating that the ordered crystalline structure with the dendronized alkyl chains could promote the charge carrier transport. This paper could intensify the relationship between the self-assembly structure with charge carrier mobilities and strengthen the potential applications of SPDIs in organic field-effect transistors.

## Data Availability

Data can be obtained on request from the corresponding author.

## Supplementary Materials

The following supporting information can be downloaded at: https://www.mdpi.com/article/10.3390/molecules29091964/s1, Table S1: Crystallographic parameters of linear SPDI; Figure S1. DFT-optimized molecular conformations of (a) linear SPDI and (b) dendronized SPDI, and the calculated intermolecular distance of (c) linear SPDI (3.4878 Å) and (d) dendronized SPDI (3.1437 Å); Figure S2. (a) Model of the helical column in the monoclinic lattice of linear SPDI; (b) high-resolution STM image of linear SPDI; Figure S3: ^1^H NMR spectrum of compound **2**; Figure S4: ^1^H NMR spectrum of linear SPDI; Figure S5: ^1^H NMR spectrum of dendronized SPDI.
