# Supplementary material for: Self-Assembly Behavior, Aggregation Structure, and the Charge Carrier Transport Properties of S-Heterocyclic Annulated Perylene Diimide Derivatives
Source: Molecules. 2024 Apr 25;29(9):1964. doi: 10.3390/molecules29091964 (PMC11085381; doi:10.3390/molecules29091964)
Supplement: Supplementary file 1 [file molecules-29-01964-s001.zip › molecules-2950394-supplementary.pdf]

Supplement Files

Self-Assembly Behavior, Aggregation Structure, and the Charge Carrier Transport Properties of S-Heterocyclic Annulated Perylene Diimide Derivatives

Haijie Ben [1], Gaojie Yan [2], Yulin Wang [1], Huiming Zeng [1], Yuechao Wu [1], Feng Lin [1], Junhua Zhao [1], Wanglong Du [3], Shaojie Zhang [1], Shijia Zhou [1], Jingyu Pu [1], Milan Ye [1], Haifeng Ji [2,*] and Liang Lv [1,*]

[1] College of Chemical and Material Engineering, Quzhou University, Quzhou 324000, China; benhaijie@tju.edu.cn (H.B.); csu_lin@163.com (Y.W.); weimingzeng@tom.com (H.Z.); wuyuechao_qzu@163.com (Y.W.); linf2015@126.com (F.L.); qzzjh@qzc.edu.cn (J.Z.); zhangshaojie031026@163.com (S.Z.); yemilan@qzc.edu.cn (M.Y.)

[2] Shenzhen Research Institute of Nankai University, Nankai University, Shenzhen 518083, China; 202021501025@stu.hebut.edu.cn

[3] College of Chemical Engineering, Zhejiang University of Technology, Hangzhou 310014, China

* Correspondence: haifengji@nankai.edu.cn (H.J.); lianglv@qzc.edu.cn (L.L.)

1. DFT-optimized molecular conformations and the calculated intermolecular distance of linear SPDI and dendronized SPDI

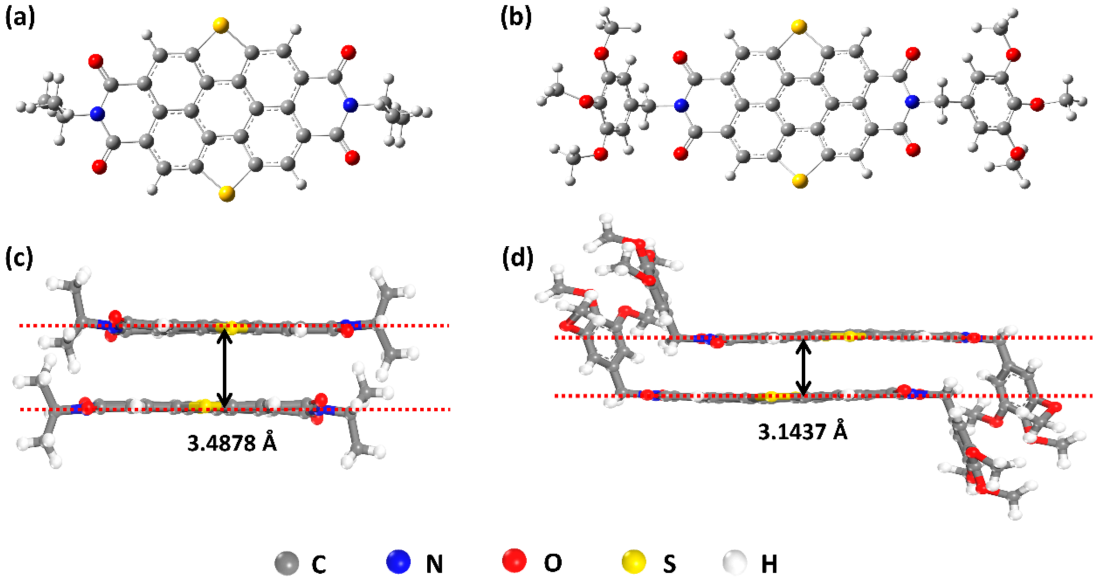

Figure S1. DFT-optimized molecular conformations of (a) linear SPDI and (b) dendronized SPDI, and the calculated intermolecular distance of (c) linear SPDI (3.4878 Å) and (d) dendronized SPDI (3.1437 Å).

2. The crystallographic parameters of linear SPDI

Table S1. Crystallographic parameters of linear SPDI

	2θ (deg)		*d*-spacing (nm)		Intensity
(*hkl*)	expt[a]	calcd[b]	expt[a]	calcd[b]	expt[c]
100	2.80	2.80	3.16	3.16	w
010	3.00	3.00	2.95	2.95	vs
-110	3.75	3.75	2.36	2.36	vw
020	6.00	6.00	1.47	1.47	s
-220	7.50	7.50	1.18	1.18	vw
030	9.00	9.01	0.98	0.98	s
-330	11.00	11.27	0.80	0.79	vw
060	18.00	18.07	0.49	0.49	w
540	20.00	19.99	0.44	0.44	m
630	20.70	20.43	0.43	0.43	w
640	22.00	22.34	0.40	0.40	w
650	23.60	24.48	0.38	0.36	m
660	26.00	26.81	0.34	0.33	m
011	8.60	8.92	1.03	0.99	m
021	9.90	10.33	0.89	0.86	m
-321	12.70	12.69	0.70	0.70	m
-331	14.00	14.07	0.63	0.63	m
331	15.80	15.76	0.56	0.56	w
-341	16.50	15.91	0.54	0.56	w
541	21.00	21.71	0.42	0.41	w
022	17.00	17.90	0.52	0.50	w
032	18.80	19.13	0.47	0.46	vs
033	25.60	26.98	0.35	0.33	w

3. The direct image of self-assembly of linear SPDI

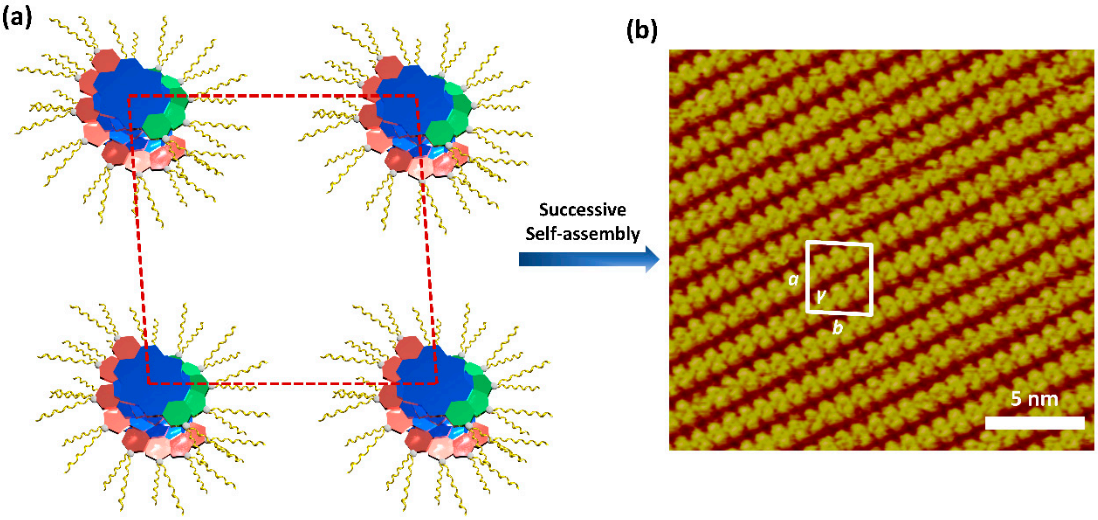

Figure S2. (a) Model of the helical column in the monoclinic lattice of linear SPDI; (b) high-resolution STM image of linear SPDI.

4. The ^1H NMR spectrum of compound 2 recorded in $CDCl_3$

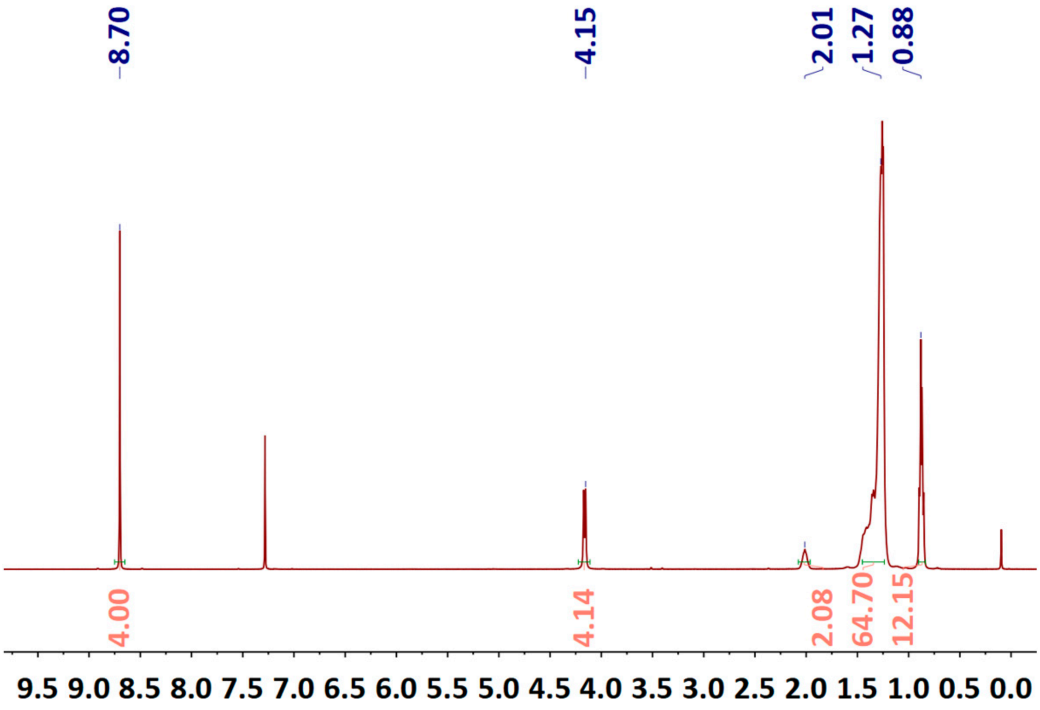

Figure S3. ^1H NMR spectrum of **compound 2**.

5. The ^1H NMR spectrum of linear SPDI recorded in $CDCl_3$.

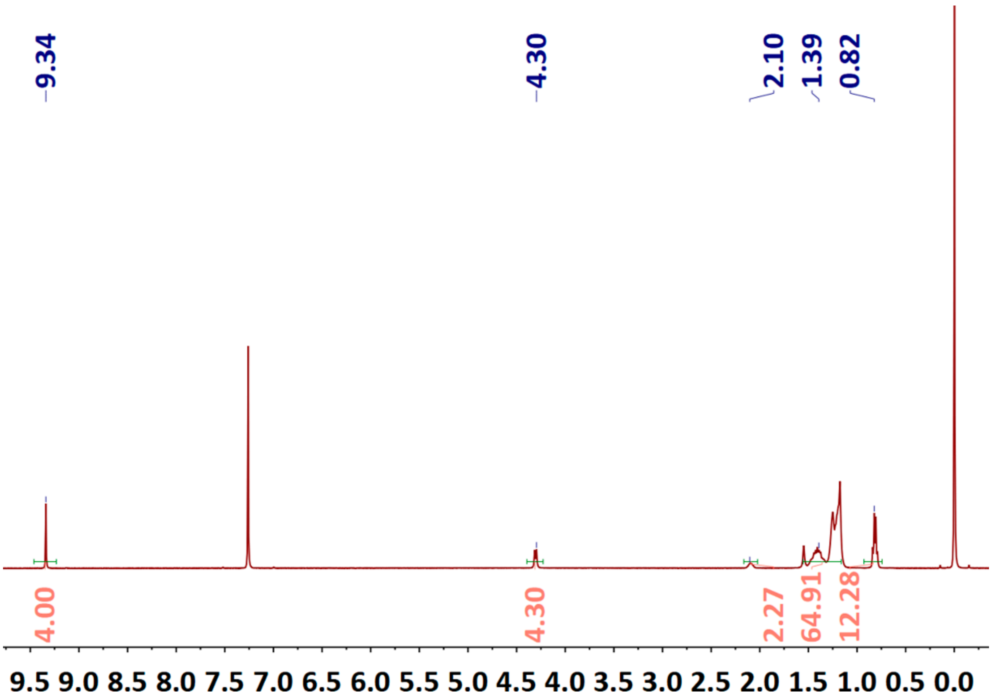

Figure S4. ^1H NMR spectrum of **linear SPDI**.

6. The ^1H NMR spectrum of dendronized SPDI recorded in $CDCl_3$.

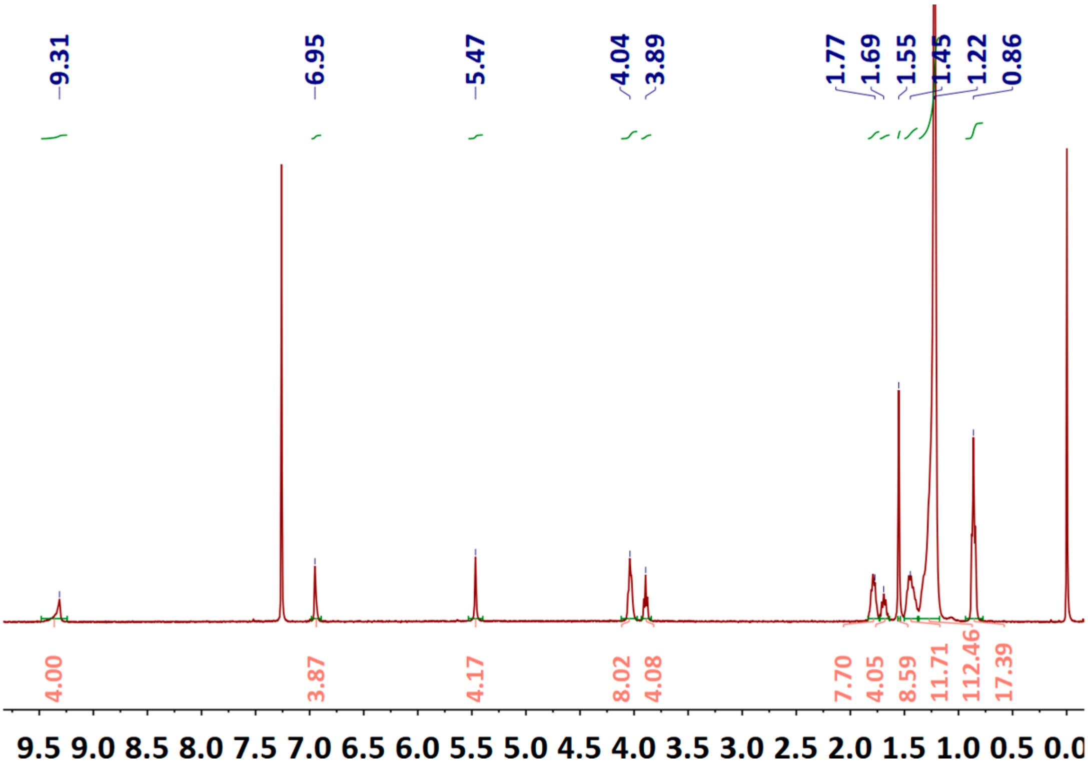

Figure S5. ^1H NMR spectrum of dendronized SPDI.
